# A Medical Causerie

**Published:** 1915-10-09

**Authors:** 


### A MEDICAL CAUSERIE.

The opening of the medical session has been distinguished neither by addresses nor by dinners. Which of the two has been the more acutely missed is hardly a difficult question to answer. In one sense neither has been missed, for the times furnish other food for thought. Yet when peace comes again the dinners will certainly reappear, for they furnish occasions, otherwise non-existent, for the meetings of old friends and for the cultivation of school loyalty, which, though sometimes unduly strident, is not without its pleasing side. But the opening addresses are possibly in peril, for both speakers and audiences are not free from a sense of relief. It is not given to every medical teacher, apparently indeed to only a few of them, to escape the platitude and the commonplace which so readily abound at these times, and students may find other occasions for a display of high spirits. When the exceptional man can be found by all means let him be encouraged, but having discovered that we can make a good start without the infliction of dulness and stale quotations we shall be reluctant to return to these exercises. Were all the London schools to arrange a joint meeting to be addressed by some really inspiring personality the effect would be impressive on both the audience and the public, and the occasion would justify itself. But even among this select group there are Jews who have no dealings with the Samaritans, and the more feeble folk feel they must assert what individuality they possess.

The proverbial difficulties of definition have brought to nought one of the Treasury proposals, and so, after all, "hats" may continue to enter the country duty free. To define a "hat" sounds a simple task, and yet it has proved in practice to be insuperable. Lord Morley once said that while we all know an elephant when we see one we should have great difficulty in defining the animal; and a "hat" apparently comes under the same law. Not so many years ago a tax on hats might have excited some hostility in the medical profession, but just now—such, at least, is the fond belief of the Chancellor of the Exchequer—we are all patriots and are begging to be taxed. And, truth to tell, we are not so particular about our hats as we used to be. Time was when the silk hat was the mark of even the most humble practitioner. Nor was its value merely ornamental. For in those far-off times a wooden stethoscope suspended in the crown, or fixed by a forcible twist into the oblique diameter, was an equipment with which the practitioner sallied forth prepared to meet all emergencies. Something more is needed nowadays, and the handbag, which was once the signal of those on obstetric business bent, has become almost universal. The flexible stethoscope does not propose the silk hat, but naturally seeks accommodation in the bag or pocket. Here is probably one of the reasons why a less dignified headgear has become so popular in the profession. But still more powerful has been the advent of the motor-car. And the effect of this on professional dress has not been limited to headgear. It has helped to do away with the stiff formality of the frock-coat, and has led to a sartorial carelessness which would have excited horror in our forefathers. Yet we may hope to be none the worse practitioners even though we are not modelled as the fashion-plates. Less excuse can be offered for the cigarette habit, which must often be disagreeable to patients. The old custom used to be to abstain from smoking until the round of visits was over, but nowadays one sees the cigarette in full blast even while the doctor is waiting on the patient's door-step.

The secretaries of societies and sections have another trying winter in front of them. To judge from the programmes that have been issued they are full of hope, but this, it is to be feared, will be but scantily realised. What with the distractions of the hour, the absence of men at the Front, and the pressure on those who remain at home, only a scanty crop of original contributions can be expected from the world of medicine. Last winter was bad enough, but the medical societies are likely to fare even worse in the session which is just ahead of us. When the war is over there will doubtless be much attractive material for papers and discussions, and we may expect additions to our knowledge both of medicine and surgery. In this respect it is noteworthy the number of valuable observations which have been recorded by men engaged on active service. Particularly is this true in relation to injuries to the central nervous system, and here, not only medicine and surgery, but also physiology is likely to be advanced. Medical practice, of course, owes much to the men of science, but sometimes the debt is in the other direction. Disease or injury may make an experiment not possible in the laboratory, and if keen eyes and informed observation are at hand to read the message, the physiologist may learn from the practitioner a truth which had hitherto escaped him.

It is not quite certain at the moment whether medical practitioners will be called on to pay the full 3d. additional duty which is to be imposed on each gallon of petrol. There is a natural reluctance just now to do anything which looks like an attempt to avoid our share of the national charges, and the profession will accept the verdict of the authorities without demur, whatever it be. But the Chancellor has deliberately left the question open for discussion when the Finance Bill comes before the House, and the case for the profession will then be represented. Sir Henry Craik, who raised the question in Parliament, is always alert when medical interests are at stake. He is the member for the Universities of Glasgow and Aberdeen, and consequently medical graduates form a large part of his constituency. Hence he is kept well posted in matters professional.

